# Development of catecholaminergic neurons of Otp-lineage in the medial extended amygdala and related forebrain centers

**DOI:** 10.3389/fnana.2025.1553952

**Published:** 2025-03-19

**Authors:** Lorena Morales, Ester Desfilis, Loreta Medina

**Affiliations:** ^1^Department of Medicina Experimental, Universitat de Lleida, Lleida, Spain; ^2^Laboratory of Evolutionary Developmental Neurobiology, Lleida’s Institute for Biomedical Research-Dr. Pifarré Foundation (IRBLleida), Lleida, Spain

**Keywords:** catecholamines, tyrosine hydroxylase, medial extended amygdala, medial bed nucleus of the stria terminalis, preoptic area, hypothalamus

## Abstract

Catecholaminergic (CA) neurons of the medial extended amygdala, preoptic region and adjacent alar hypothalamus have been involved in different aspects of social behavior, as well as in modulation of homeostasis in response to different stressors. Previous data suggested that at least some CA neurons of the medial extended amygdala could originate in a hypothalamic embryonic domain that expresses the transcription factor Otp. To investigate this, we used Otp-eGFP mice (with permanent labeling of GFP in Otp cells) to analyze coexpression of GFP and tyrosine hydroxylase (TH) throughout ontogenesis by way of double immunofluorescence. Our results provide evidence that some forebrain CA cells belong to the Otp lineage. In particular, we found small subpopulations of TH cells that coexpress GFP within the medial extended amygdala, the periventricular preoptic area, the paraventricular hypothalamus, the periventricular hypothalamus, as well as some subdivisions of the basal hypothalamus. In some of the Otp cells, such as those of extended amygdala, the expression of TH appears to be transitory, in agreement with previous studies. The results open interesting questions about the role of these Otp versus non-Otp catecholaminergic subpopulations during development, network integration and in modulation of different functions, including homeostasis and social behaviors.

## 1 Introduction

Some of the forebrain areas that comprise the social brain network (SBN) contain subpopulations of catecholaminergic (CA) neurons (Tillet, 1994; Northcutt and Lonstein, 2011; Huang et al., 2020), including the medial preoptic area (MPO), the paraventricular and supraoptic nuclei (Pa or PVN, and SO, respectively), and the medial extended amygdala (EAme). CA neurons of the hypothalamus have also been involved in stress-induced modulation of homeostasis by way of projections to the median eminence, and some also appear to modulate autonomic function by way of descending projections to the dorsomedial medulla, including the dorsal vagal complex, and the spinal cord (Swanson and Sawchenko, 1980; Kvetnansky et al., 2009; Zhao et al., 2011; Grassi et al., 2022). These CA cells of the forebrain have been classified in two major groups, known as A15 and A14. The A15 group includes cells located in the bed nucleus of the stria terminalis (BST), as well as other cells mainly located in the preoptic area. The CA cells of A14 are primarily found across the alar and basal hypothalamus (Tillet, 1994; Marín et al., 2005; Björklund and Dunnett, 2007). However, this alphanumerical system does not reflect the great heterogeneity of CA cells based on their spatial locations in the forebrain (Bilbao et al., 2022), and their transcriptional, morphological and physiological features (Romanov et al., 2017, 2020; Korchynska et al., 2022). In addition, this classification excludes other described CA cells (Marín et al., 2005), such as those present in the medial amygdalar nucleus (Northcutt et al., 2007). The cell abundance in the latter nucleus, medial BST and preoptic area varies between different mammalian species and sexes (Northcutt et al., 2007; Simerly et al. (1985a); based on immunohistochemical detection of the enzyme tyrosine hydroxylase or TH), but also with changes in circulating sex steroids (for example, in relation to the hormone surge in female rats during estrous, or after partum in female mice), with the social context (such as following exposure to social novelty), as well as with the age and the stage of development (Simerly et al., 1985b, Simerly, 1989; Northcutt et al., 2007; Cavanaugh and Lonstein, 2010a, 2010b; Bupesh et al., 2014; Scott et al., 2015; Ugrumov, 2024; based on protein and mRNA detection of TH).

An intriguing aspect of these TH-positive (TH+) cells is that many of them do not express other enzymes for the synthesis of catecholamines, such as the aromatic amino acid decarboxylase (AADC), necessary for synthetizing dopamine, meaning that they produce just L-DOPA, and some only express TH mRNA but not the protein (Ugrumov et al., 2002; Ahmed et al., 2012; Bupesh et al., 2014; Ugrumov, 2024), raising additional questions on their ‘catecholaminergic nature’ and their function (Björklund and Dunnett, 2007). Nevertheless, there is evidence that L-DOPA can act as neurotransmitter or neuromodulator, both in the striatum and hypothalamus (Misu et al., 2002; Ugrumov, 2009). Moreover, it appears that the protein and, at lower level, the mRNA expression of TH, the rate-limiting enzyme for the synthesis of catecholamines, is transient, although dynamic (increasing in certain contexts) in some cells of the medial extended amygdala, preoptic area and hypothalamus of species such as mice and rats (Northcutt et al., 2007; Bupesh et al., 2014; De Guzman et al., 2023), raising questions on the role of this transient and dynamic expression.

Functionally, the prosencephalic CA cell populations of the SBN present multiple roles in both development and adulthood. On the one hand, catecholamines act as morphogens during different moments of development (reviewed by Lauder, 1988, 1993; Ugrumov, 2024). In mouse embryo, catecholaminergic release regulates maturation of their target neurons (Lauder, 1993). In rats, the hypothalamic catecholaminergic systems play a critical role in controlling the phenotype of target neurons, modulating their gene expression, axonal growth, and neurotransmitter specific synthesis, uptake, and release (Ugrumov, 1997, 2024). On the other hand, in the adult brain they become active during different aspects of social behavior, including aggression, affiliation, and sexual behaviors (Bell and Hepper, 1987; Pfaus et al., 1990; Hull et al., 1993; Gobrogge et al., 2007; Yanowitch and Coccaro, 2011; Eiden, 2013; Hiura et al., 2018). Moreover, TH+ cells of the periventricular preoptic area are sexually dimorphic in rat and mouse, and are more abundant in females, especially after partum (Simerly et al., 1985a; Scott et al., 2015; based on TH immunohistochemical detection). Moreover, CA neurons of the medial extended amygdala, preoptic region and alar hypothalamus play an important role in monogamous animals, being involved in most, if not all, of the key cognitive and behavioral processes associated with pair bonding (Wang et al., 1997; Cushing et al., 2003; Lim and Young, 2004; Young and Wang, 2004; Hull and Dominguez, 2006; Gobrogge et al., 2007, 2017; Young et al., 2008; Northcutt and Lonstein, 2009; Cavanaugh and Lonstein, 2010a; Walum and Young, 2018; Rothwell et al., 2019).

The manifold functions of the CA neurons of the SBN are the result of the great heterogeneity of these neurons, which appears to arise during development as different neuron subsets originate from molecularly distinct progenitors, expressing specific combinations of transcription factors (Romanov et al., 2020). Despite the extensive knowledge about the location and functions of CA neurons of the SBN, the embryonic origin and other details of the development of the different subpopulations remain poorly understood, although a few aspects are known, as explained next. Based on the widespread distribution and prosomeric organization of the different CA cell groups (Foster, 1994; Smeets and Reiner, 1994; Smeets and González, 2000; Bilbao et al., 2022), it is likely that there are multiple origins, involving different progenitor pools, as proposed previously (Puelles and Medina, 1994). Different types of results support this idea, including fate-mapping assays (for example, Yun et al., 2003; Bupesh et al., 2014). In addition, single-cell transcriptome in mouse throughout development points to the existence of at least 10 different dopaminergic cell clusters only in the hypothalamus, coexpresing the mRNA of TH, AADC (Ddc) and vesicular monoamine transporter (VMAT2 or Slc18a2) (Romanov et al., 2020). Many of these dopaminergic cells, including those of the preoptic region, appear to develop from intermediate progenitors expressing the transcription factor Ascl1 (Achaete-Scute Family BHLH Transcription Factor 1), which cooperates with Isl1 (Islet1), Dlx and other transcription factors (TFs) to define cell identity (Romanov et al., 2020; Zhang et al., 2021). Many TH+ cells derived from Ascl1 progenitors are GABAergic, based on coexpression with GAD1 and/or GAD2 and other GABAergic markers (Romanov et al., 2017, 2020; Zhang et al., 2021; based on mRNA detection). However, coexpression of TH with GABAergic markers changes depending on the preoptic or hypothalamic subdivision (Negishi et al., 2020; based on immunoreactivity for TH). This means that some TH+ cells of the preoptic region and hypothalamus are non-GABAergic and might derive from progenitors producing non-GABA cells. This is in line with findings suggesting that TH+ cells of the paraventricular hypothalamic nucleus and medial extended amygdala might originate in the supraopto-paraventricular domain of the hypothalamus (SPV), expressing the transcription factor Otp during development (Bupesh et al., 2014). Recent studies showed that Otp is expressed by a second subtype of intermediate progenitors of the hypothalamus, which also expresses Neurog2, and is found in complementary domains to those containing the Ascl1 intermediate progenitors (Zhang et al., 2021). The Neurog2/Otp intermediate progenitors give rise to different subsets of immature postmitotic cells, some of them also expressing Otp and/or Sim1, that finally produce glutamatergic cells also containing different neuropeptides, such as vasopressin (AVP), oxytocin (OXT), corticotropin-relseasing factor (CRF) or tyrotropin-releasing factor (TRH) (Zhang et al., 2021; based on mRNA detection). These neuropeptidergic cell populations do not form in the absence of Otp (Wang and Lufkin, 2000; Acampora et al., 2000). Single-cell transcriptome in mouse showed that OXT and AVP cells of the SPV express the mRNA of TH and the glutamatergic marker Slc17a6/VGLUT2 at very low levels (Romanov et al., 2017). This agrees with some studies showing coexpression of AVP and OXT with TH in the human hypothalamic paraventricular and supraoptic nuclei (Panayotacopoulou et al., 1994, 2000; Kontostavlaki et al., 2007; based on immunoreactivity for TH). Coexpression of AVP and TH has also been found in these hypothalamic nuclei in tree shrews (Huang et al., 2020; based on TH immunoreactivity). In zebrafish, Otp is essential for the development of TH+ cells of the neurosecretory preoptic nucleus and those of the A11 CA group, located in the posterior tubercle (Ryu et al., 2007; Blechman et al., 2007). All of these data point to Otp as an important TF for the development of some CA cell supopulations, and support the idea that, in mammals, a subset of TH+ neurons of the medial extended amygdala and hypothalamus might derive from Otp-lineage cells, as suggested previously (Bupesh et al., 2014). To check this, we took advantage of an Otp reporter mouse line (Otp-eGFP), with labeling of enhanced GFP in Otp cells, to analyze coexpression of GFP with TH in areas of the SBN at embryonic, prepuberal and adult ages. The Otp-eGFP has been previously validated by studying coexpression of GFP and Otp in forebrain cells (Morales et al., 2021), and by comparing it with an Otp-Cre mouse line (Morales et al., 2022), showing that GFP recapitulates the known pattern of Otp expression in the forebrain during development, and that this reporter protein remains visible in Otp cells throughout ontogenesis.

Our results show co-expression of TH and GFP (Otp-related) in subsets of CA cells of the medial bed nucleus of the stria terminalis, the sexually dimorphic periventricular preoptic area, the paraventricular hypothalamic nucleus (dorsal/anterior and central parts), periventricular hypothalamus, supraoptic hypothalamic nucleus, and some subdivisions of the basal hypothalamus. Notably, the presence of double-labeled cells in BSTM appeared to be transient. This raises questions on the role of TH in Otp and non-Otp cells during the development of specific functional subcircuits involved in homeostasis and/or social behavior control.

## 2 Materials and methods

### 2.1 Experimental animals

For the present study, we employed Otp-eGFP knockin transgenic mice (*Mus musculus*, Tg (Otp-EGFP) OI121Gsat/Mmucd; Mutant Mouse Resource & Research Centers, MMRRC supported by NIH, University of California at Davis, USA), including embryos of 18.5 days of development (post-coitum; E18.5) (*n* = 6, 3 females and 3 males), prepuber postnatals of 19 days (P19) (*n* = 6, 3 females and 3 males) and adults of 100 days (P100) (*n* = 4, 2 females and 2 males). The genotype and sex of the animals were determined by means of PCR at the Proteomics and Genomics Service of the Biomedical Research Institute of Lleida (IRBLleida).

This transgenic mouse line was kept in the pathogen-free area, which fulfills all requirements for genetically modified animals (notification no. A/ES/19/I-06) of the rodent animal facility of the University of Lleida (REGA license no. ES251200037660). The adult animals and weaned-off postnatal were housed in groups of three to five animals, at 22 ± 2°C on a 12 h light/dark cycle, with free access to food and water. All the animals were treated according to the regulations and laws of the European Union (Directive 2010/63/EU) and the Spanish Government (Royal Decree 53/2013 and 118/2021) for the care and handling of animals in research. All the protocols used were approved by the Committees of Ethics for Animal Experimentation and Biosecurity of the University of Lleida, and by Generalitat de Catalunya (Authorization no. 10038).

### 2.2 Tissue collection and fixation

At appropriate development days, the mouse embryos were obtained by cesarean section from pregnant females, which were previously sacrificed by a lethal dose of sodium pentobarbital (0.1 mg/g; i.p.). Perinatal embryos and postnatal animals (E18.5 to P100) were deeply anesthetized with sodium pentobarbital (0.1 mg/g; i.p.) and then transcardially perfused with 0.9% saline solution (0.9% NaCl), followed by 4% PFA. After dissection, the brains were postfixed by immersion in 4% PFA overnight at 4°C.

### 2.3 Sample preparation and sectioning

Embryonic brains processed for immunofluorescence were previously cryoprotected by maintaining them overnight within a solution of 30% sucrose in saline phosphate buffer (PBS 0.1 M; pH = 7.4) at 4°C. Then, they were embedded in a block of 20% gelatin diluted in 30% sucrose. The block was fixed with 4% PFA in 30% sucrose overnight at 4°C and sectioned with a freezing microtome (Microm HM 450, Thermo Fisher Scientific, United Kingdom) on the transversal and sagittal planes at 18 or 40 μm of thickness and collected in cold PBS.

Postnatal brains for either immunohistochemistry or immunohistofluorescence were cryoprotected by immersion in a solution of 10% glycerol and 2% DMSO in phosphate buffer (PB; pH = 7.4) for 2 days, followed by immersion in a solution of 20% glycerol and 2% DMSO in PB for 3 days (Rosene et al., 1986), after which they were frozen in −60/−70°C isopentane (2-methyl butane, Sigma-Aldrich, Germany) with dry ice for about 1 min and preserve at −80°C until use. Frontal or sagittal free-floating sections of 60 μm of thickness were obtained using a freezing microtome (Microm HM 450, Thermo Fisher Scientific, United Kingdom), collected in cold PBS.

### 2.4 Double immunofluorescence

After tissue permeabilization and blocking of non-specific binding sites (as previously described in Morales et al., 2021), the sections were incubated with a cocktail of the primary antibodies, chicken anti-GFP and rabbit anti-TH (see Table 1), diluted in PBS-Tx for 72 h at 4°C and gentle agitation. Following this, sections were washed and then incubated for 90 min at RT, in a cocktail of fluorescent secondary antibodies, goat anti-chicken Alexa 488, and donkey anti-rabbit Alexa 568 (Table 2), diluted in PBS-Tx. Finally, the sections were rinsed, mounted using 0.25% gelatin in Tris buffer (TB; pH = 8; 0.1 M) and coverslipped using an antifading mounting medium (Vectashield Hardset Antifade mounting medium).

**Table 1:** Primary antibodies employed.

Type	Antibody	Antigen recognized	Immunogen	Dilution	Manufacturer and Reference	RRID
Polyclonal	Chicken anti-GFP, IgY	Green fluorescent protein (GFP)	Recombinant full-lenght protein corresponding to GFP	1:1000	Abcam Antibodies, Ref. ab13970	AB_300798
	Rabbit anti-TH	Tyrosine hydroxylase	Denatured tyrosine hydroxylase from rat pheochromocytoma (denatured by sodium dodecyl sulfate).	1:1000	Millipore, Ref. AB152	AB_390204

**Table 2:** Secondary antibodies employed.

Type	Antibody	Dilution	Manufacturer and Reference
Fluorescent	Goat anti-chicken IgY (H + L) Alexa 488	1:500	Invitrogen, Ref. A-11039
	Donkey anti-rabbit IgG (H + L) Alexa 568	1:500	Invitrogen, Ref. A-10042

See Table 1 for a list of all primary antibodies employed. All antibodies were validated on Western blots by the respective manufacturers and produced specific staining patterns identical to those observed using *in situ* hybridization, as explained next.

The chicken anti-GFP antibody recognized a single band of 25 KDa on Western blots of HEK293 transfected cell lysates, and a band at the same molecular weight on Western blots of transgenic mouse spinal cords (data sheet of the manufacturer). No staining was seen in non-transfected cells. This antibody has been successfully used to detect enhanced GFP in Viaat-eGFP knockin transgenic mice (Aresh et al., 2017).

The rabbit anti-TH antibody recognizes denatured tyrosine hydroxylase from rat pheochromocytoma, and by Western blotting it recognizes a band of approximately 62 kDa on PC12 lysates, which corresponds to the enzyme tyrosine hydroxylase (data sheet of the manufacturer). In the developing mouse brain, it produces a staining pattern identical to that observed in previous reports in mouse, rat, and other mammals (Smeets and Reiner, 1994; Jacobowitz and Abbott, 1997), and the distribution of immunoreactive perikarya is generally identical to that observed by *in situ* hybridization in the mouse brain (Marín et al., 2005; Bupesh et al., 2014).

### 2.5 Digital photographs and figures

Serial images from fluorescent material were taken with a confocal microscope (Olympus FV1000, Olympus Corporation, Japan). Selected digital immunohistochemical images were adjusted for brightness and contrast with Adobe Photoshop 2021. Finally, the figures were mounted using CorelDRAW 2019 while the fluorescent ones were adjusted and extracted using Olympus FV10-ASW 4.2 Viewer (Olympus Corporation). The schemes (or drawings) included in the figures were made by means of CorelDraw 2012, 2019, and 2024 based on microphotographs of selected immune stained sections at representative brain levels.

### 2.6 Nomenclature

For embryonic domains and axis, we follow the prosomeric model (Puelles and Rubenstein, 2015) as well as previous studies of our group on forebrain development in mouse (Bupesh et al., 2014, on the development of TH+ cells; Morales et al., 2021, defining a new embryonic domain adjacent to the boundary between telencephalon and hypothalamus). This implies using the terms ‘dorsal’ and ‘ventral’ following topological coordinates (Nieuwenhuys and Puelles, 2016), which in the forebrain are about 90 degrees shifted with respect to the classical topographic terms. For example, the dorsal part of the paraventricular hypothalamic nucleus as used here corresponds to the anterior part of this nucleus in classical nomenclature. We did this when referring to general regions and divisions, but for specific nuclei and areas within them, we employed the terminology of the Franklin and Paxinos mouse brain atlas.

## 3 Results

We analyzed the coexpression of GFP with TH in the secondary prosencephalon of Otp-eGFP mice at E18.5 (a preterm embryonic age, when all described TH cell populations are found; Bupesh et al., 2014), at postnatal day 19 (P19) and in adulthood (P100). The selection of ages was based on the fact that some of the TH+ cell populations found in the extended amygdala at E18.5 are transient and appear to downregulate TH expression after birth (discussed in Bupesh et al., 2014); thus, we aimed to analyze these cells before and after this critical moment. We paid particular attention to the areas of the SBN where TH and GFP cells overlap. These include the medial extended amygdala and adjacent preoptic region, parts of the alar hypothalamus (including the periventricular, paraventricular and supraoptic nuclei), and parts of the basal hypothalamus (including the dorsomedial hypothalamic nucleus).

### 3.1 Medial extended amygdala and preoptic plus subpreoptic regions

#### 3.1.1 Medial extended amygdala (EAme)

**Figure 1:**
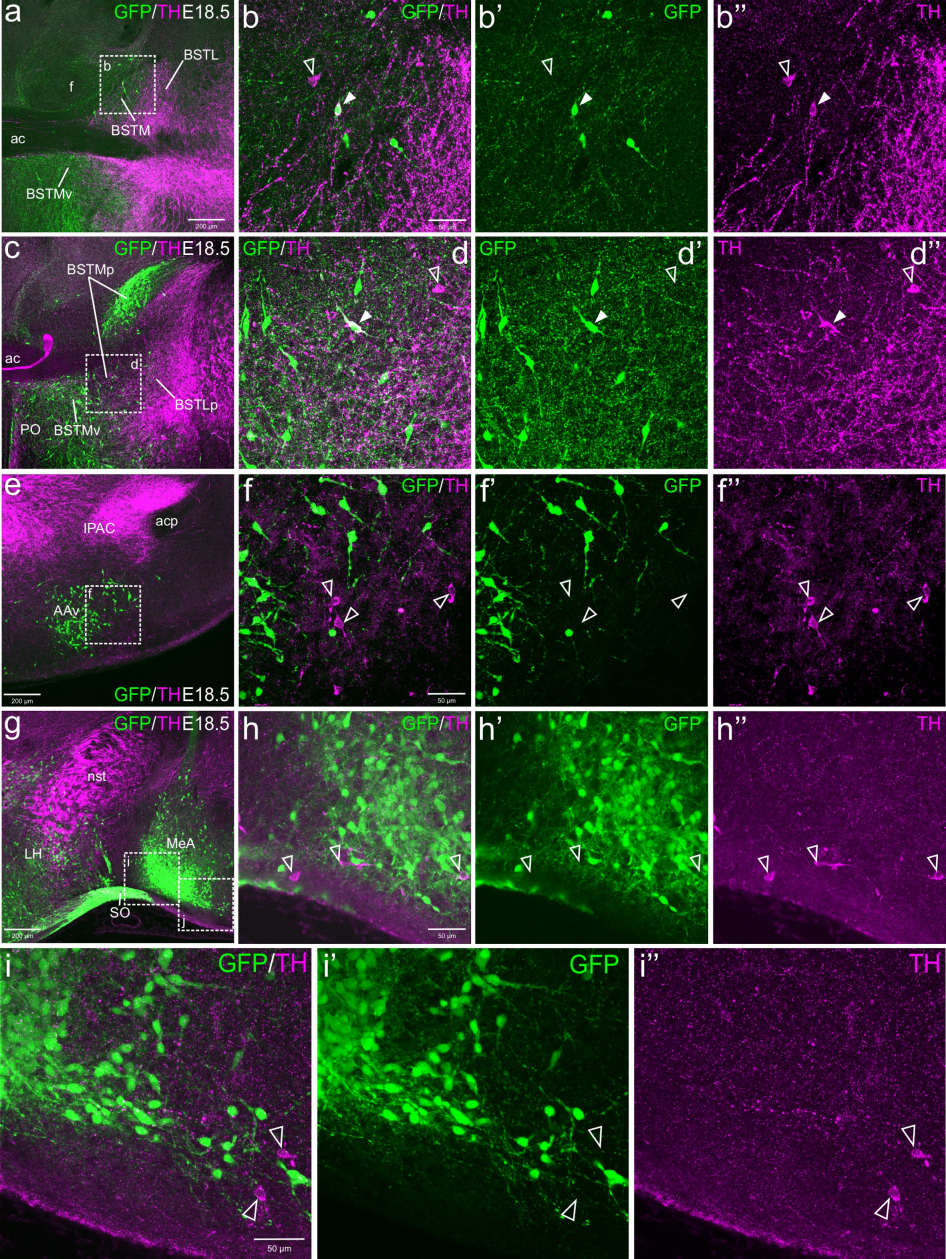
Confocal images of frontal sections of Otp-eGFP embryo brains at E18.5, double labeled for GFP (green) and TH (magenta) (using double immunofluorescence for GFP and TH), at different levels of the EAme, including commissural **(a–b”)** and postcommissural **(c–d”)** BSTM, anterior amygdaloid area **(e–f”)** and anterior part of the medial amygdala **(g–i”)**. Cells coexpressing TH and GFP are seen in posterior BSTM (filled arrowheads in **b–b”** and **d–d”**), but this and other subdivisions of EAme also contain TH cells that do not coexpress GFP (empty arrowheads in **b–b”**, **d–d”**, **f–f”**, **h–h”**). A few TH cells not coexressing GFP are also found in the anterior cortical amygdala (empty arrowheads in **i–i”**). See text for more details. For abbreviations see list. Scale: a = 200 μm (applies to **a, c, e, g**); b = 50 μm (applies to **b–b”**, **d–d”**, **f–f”**, **h–h”**); i = 50 μm (applies to **i–i”**).

At E18.5, we observed a few TH cells within different subdivisions of the EAme, mostly in the posterior and ventral parts of the medial bed nucleus of the stria terminalis (BSTM), the ventral part of the anterior amygdala (AAv) and the anterior subnucleus of the medial amygdala (MeA) (Figure 1). For better identification of BSTM subdivisions, we relied on comparison with our own data on expression of gonadal hormone receptors (González-Alonso et al., 2025). Some of the TH cells located in the posterior BSTM were found to co-express GFP (Figures 1a–d”), but none of those located in the ventral BSTM (BSTMv), AAv and medial amygdala did it (Figures 1e–h”, see empty arrowheads). In the posterior BSTM, we also found TH cells that did not express GFP, indicating the existence of at least two populations of catecholaminergic cells in this nucleus. At E18.5, we also observed few TH+ cells in the anterior cortical amygdalar area that did not coexpress GFP (empty arrowheads in Figures 1i–i”).

**Figure 2:**
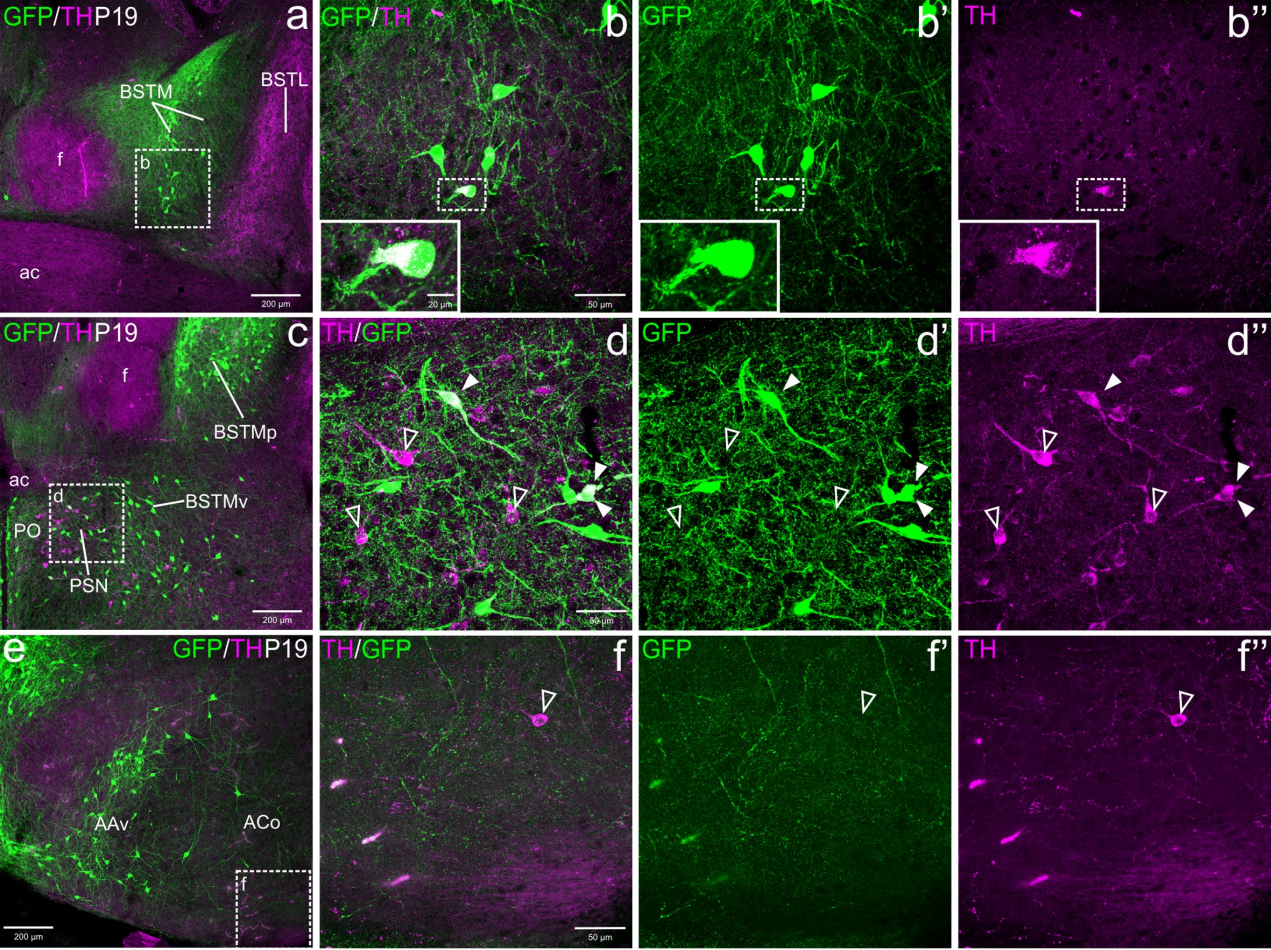
Confocal images of frontal sections of Otp-eGFP postnatal brains at P19, double labeled for GFP (green) and TH (magenta) (using double immunofluorescence for GFP and TH), at different levels of the EAme, including commissural **(a–b”)** and postcommissural **(c–d”)** BSTM and anterior amygdaloid area **(e)**. Coexpressing cells are only seen in posterior parts of BSTM (detail squared in **b–b”**). Extremely few TH cells are also seen in the anterior cortical amygdalar area, but these do not coexpress GFP **(e–f”)**. See text for more details. For abbreviations see list. Scale: a = 200 μm (applies to **a, c, e**); b = 50 μm (applies to **b–b”**, **d–d”**, **f–f”**).

**Figure 3:**
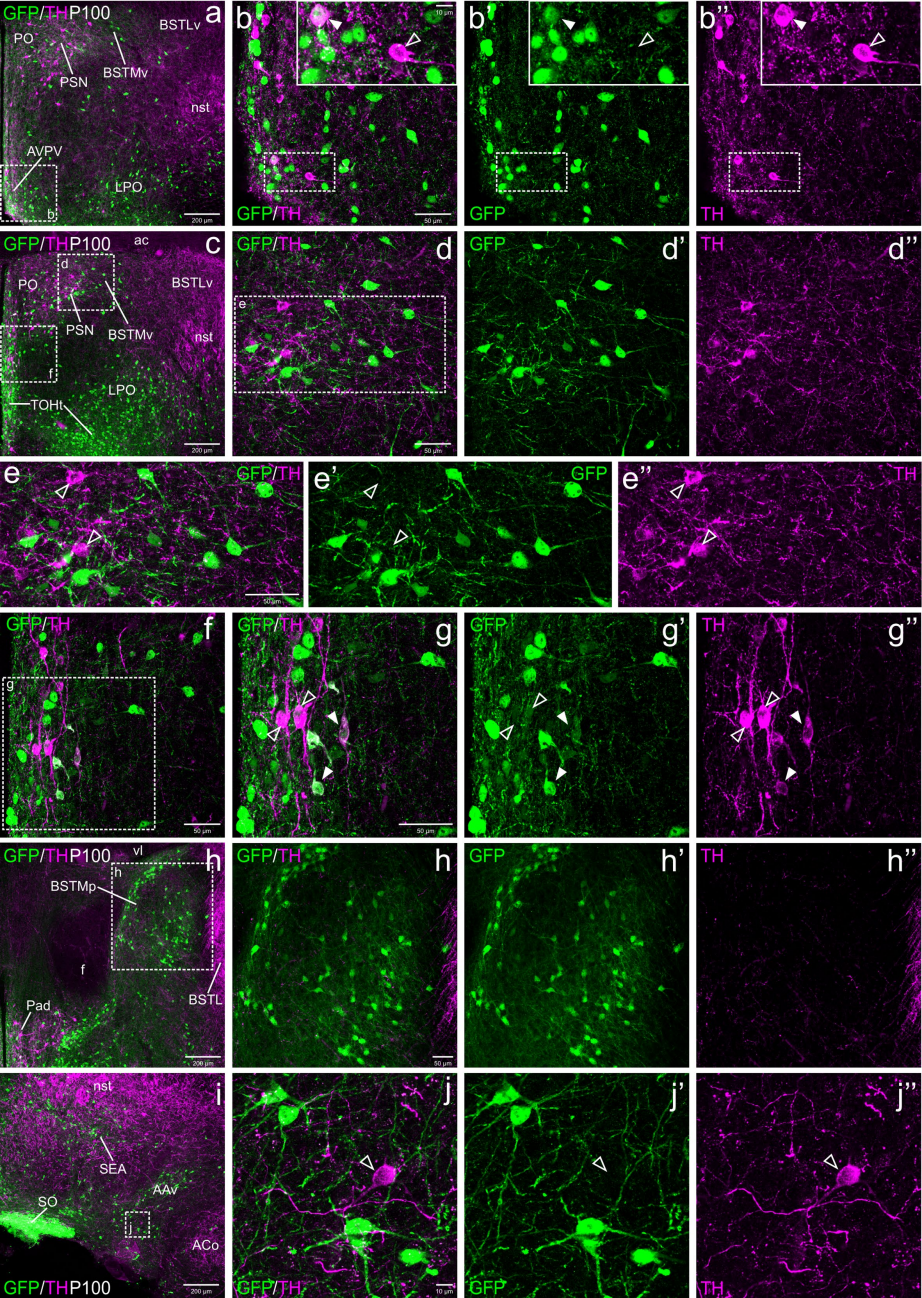
Confocal images of frontal sections of Otp-eGFP adult brains at P100, double labeled for GFP (green) and TH (magenta) (using double immunofluorescence for GFP and TH), at different levels of the EAme, including postcommissural BSTM **(h–h”)** and anterior amygdaloid area **(i–j”)**, and the preoptic area **(a–g”)**. See text for more details. In the preoptic area, cells coexpressing TH and GFP (filled arrowheads) are mostly seen in the periventricular zone, including AVPV (detail in the square of **b–b”**) and other subdivisions (detail in **g–g”**). At this age, the PSN **(a, c, d)** only includes TH cells without GFP coexpression (empty arrowheads in e–e”). For abbreviations see list. Scale: a = 200 μm (applies to **a, c, h, i**); b = 50 μm (applies to **b–b”** and **f**); d = 50 μm (applies to **d–d”**); e = 50 μm (applies to **e–e”**); g = 50 μm (applies to **g–g”**); h = 50 μm (applies to **h–h”**); j = 10 μm (applies to **j–j”**).

Postnatally, TH cells gradually disappeared in most parts of EAme, in agreement with previous studies in mouse (Figure 2). At P19, no TH+ cells were observed in MeA, but some were still present in posterior BSTM, ventral BSTMv and AAv. At this age, we could still find some TH cells in the posterior BSTM coexpressing GFP (P19: Figures 2a–b”), while none of those in BSTMv and AAv did it (P19: Figure 2e), in agreement with the situation found at E18.5. At P19, we still observed TH+ cells in the anterior cortical amygdalar area that did not coexpress GFP (Figures 2e–f”). Medial to the BSTMv, there was a distinct group of TH+ cells in the preoptic area, where we found cases of coexpression with GFP, as explained in next section (Figure 2c). In adult animals (P100), the TH cell population of BSTM was no longer observed, while in AAv it became extremely small, with cells being very scattered, and we could not find any double labeled cell (Figures 3h–j”).

#### 3.1.2 Preoptic and subpreoptic regions

From E18.5 onwards, we observed subpopulations of TH+ cells in the preoptic area (part of the subpallium) and adjacent subpreoptic region (in the terminal prosomeric part of TOH). Overlap between TH and GFP cells was mainly observed in the periventricular preoptic area, including the sexually dimorphic nucleus usually referred to as the anteroventral periventricular preoptic nucleus (AVPV; Figures 3a–b”). However, TH cells were found in the whole periventricular area, extending from the subpreoptic region (ventrally) to the juxtacommisural area, in relation to the commissural preoptic domain. Here TH cells extended laterally forming a distinct nucleus adjacent to BSTMv, which has been previously identified as the parastrial nucleus (PSN, Figures 3a,c; Simerly, 1989). All these TH subpopulations were found in similar abundance and immunoreactivity at E18.5, P19 and P100 (Figures 1c, 2c–d”, 3c–g”). Double labeling for TH and GFP showed that most of the TH cells of the preoptic area did not coexpress GFP, with the exception of some cells in the periventricular area and the PSN (filled arrowheads in Figures 2d–d”, 3d–g”). In the PSN, cases of coexpression were more frequent in younger animals (Figures 2d–d”), but they appeared to decline later, and we could not observe them in adults (Figures 3d–d” detail in e–e”). Outside the periventricular area and the PSN, extremely few cells coexpressing TH and GFP were observed in the lateral preoptic area (LPO).

### 3.2 Paraventricular nuclei, periventricular area and supraoptic nucleus of the TOH and alar hypothalamus

**Figure 4:**
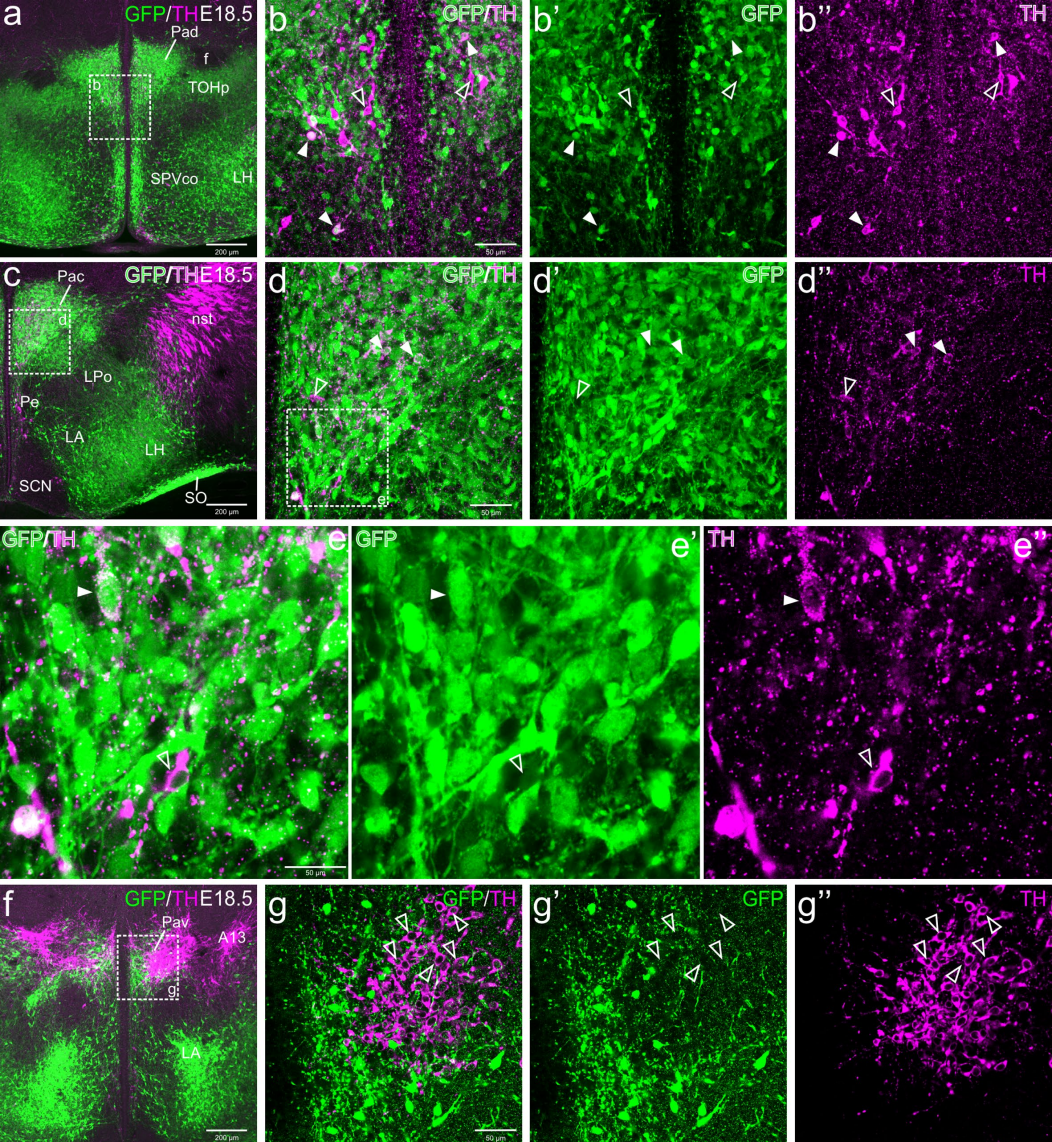
Confocal images of frontal sections of Otp-eGFP embryo brains at E18.5, double labeled for GFP (green) and TH (magenta) (using double immunofluorescence for GFP and TH), at different levels of the paraventricular nucleus, including its dorsal (Pad, **a–b”**), central (Pac, **c–e”**) and ventral (Pav, **f–g”**) subnuclei. Cells coexpressing TH and GFP are seen in Pad and Pac (filled arrowheads), but not Pav (empty arrowheads). See text for more details. For abbreviations see list. Scale: a = 200 μm (applies to **a, c, f**); b = 50 μm (applies to **b–b”**, **d–d”**, **g–g”**); e = 50 μm (applies to **e–e’**).

**Figure 5:**
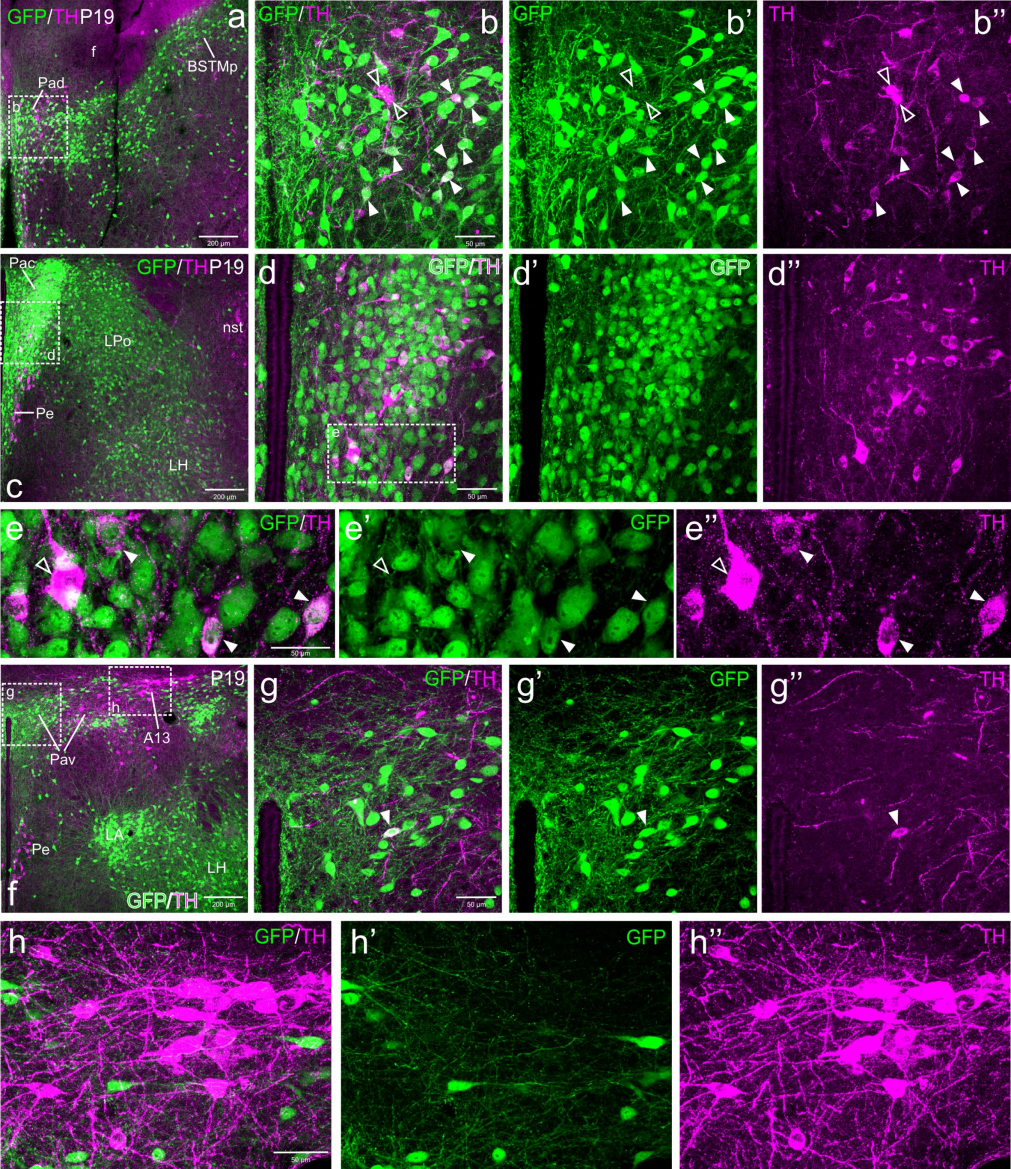
Confocal images of frontal sections of Otp-eGFP postnatal brains at P19, double labeled for GFP (green) and TH (magenta) (using double immunofluorescence for GFP and TH), at different levels of the paraventricular nucleus, including its dorsal (Pad, **a–b”**), central (Pac, **c–e”**) and ventral (Pav, **f–h”**) subnuclei. Cells coexpressing TH and GFP (filled arrowheads) are seen in Pad (details in **b–b”**) and Pac (details in **e–e”**). In Pac, most double-labeled cells are parvocellular, but this nucleus also contains magnocellular TH cells that do not coexpress GFP (empty arrowheads in **e–e”**). Close to the GFP cells of Pav, the TH+ A13 cell group is observed, but its cells do not coexpress GFP (**f, h–h”**). See text for more details. For abbreviations see list. Scale: a = 200 μm (applies to **a, c, f**); b = 50 μm (applies to **b–b”**, **d–d”**, **g–g”**); e = 50 μm (applies to **e–e”**); h = 50 μm (applies to **h–h”**).

**Figure 6:**
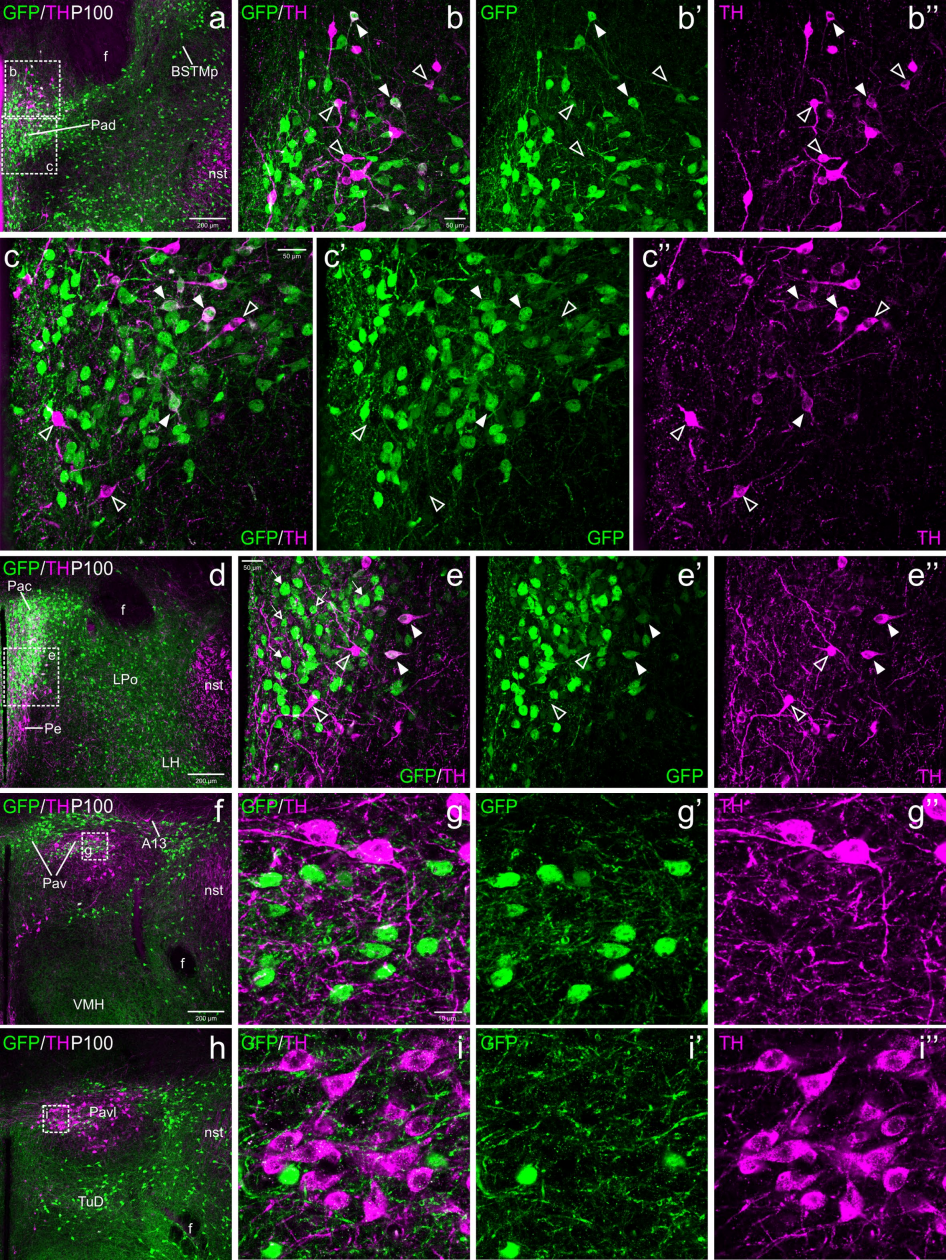
Confocal images of frontal sections of Otp-eGFP adult brains at P100, double labeled for GFP (green) and TH (magenta) (using double immunofluorescence for GFP and TH), at different levels of the paraventricular nucleus, including its dorsal (Pad, **a–c”**), central (Pac, **d–e”**) and ventral (Pav, **f–i”**) subnuclei. TH/GFP double labeled cells (filled arrowheads) are seen in Pad (details in **b–b”**, **c–c”**) and Pac (details in **e–e”**). In Pav, TH and GFP cells are mostly segregated, and its cells are adjacent to, and partially intermingled with the large TH perikarya of A13. See text for more details. For abbreviations see list. Scale: a = 200 μm (applies to **a, d, f, h**); b = 50 μm (applies to **b–b”**, **e–e”**); c = 50 μm (applies to **c–c”**); g = 10 μm (applies to **g–g”**, **i–i”**).

The paraventricular hypothalamic complex includes three major divisions along the dorsoventral axis, with the dorsal (or anterior) subnucleus belonging to the TOH and the central and ventral subnuclei being part of the core of the supraopto-paraventricular domain (SPVco) of the alar hypothalamus (Morales et al., 2021, 2022). All subdivisions are in the peduncular prosomeric parts of these domains, rich in GFP cells and contained subpopulations of TH neurons (Figures 4–6).

#### 3.2.1 Dorsal paraventricular nucleus (Pad)

TH cells in the dorsal paraventricular nucleus were found in similar abundance at E18.5 before birth (Figure 4a–b”), postnatally at P19 (Figures 5a–b”) and in adulthood (Figures 6a–c”). However, they increased their TH immunoreactivity with age. At E18.5, in general the cells presented a lumpy and weak labeling (Figures 4a–b”), while at P19 they were better defined (Figures 5a–b”) and at P100 (Figures 6a–c”) they were uniformly labeled and most showed well-defined immunoreactive dendrites. At all ages, we found that some cells within the nucleus were strongly TH immunoreactive, in contrast others showed lower immunoreactivity (Figures 4a–b”, 5a–b”, 6a–c”). From E18.5 onwards, we could observe that many of the TH cells located in the Pad co-expressed GFP, representing a bit more than one third (filled arrowheads in figures), while there were other TH cells that were not labeled for GFP (empty arrowheads in Figures 4a–b”, 5a–b”, 6a–c”). Most double labeled cells of Pad were located in ventral and lateral parts of the nucleus (Figures 4a–b”, 5a–b”, 6a–c”).

#### 3.2.2 Central paraventricular hypothalamic nucleus (Pac)

TH cells in Pac were scarce and showed very low immunoreactivity at E18.5, but they became more numerous and intensely labeled postnatally, being mostly located in ventral and lateral parts of the nucleus (Figures 4c–e”, 5c–e”, 6d–e”). Double labeling of TH and GFP at E18.5 revealed that some of the TH cells of Pac co-expressed GFP (E18.5: filled arrowheads in Figures 4c–e”). The Pac is known to contain two main morphologically, chemically and functionally different cell populations, the so-called magnocellular and parvocellular neurons (reviewed by Swanson and Sawchenko, 1980). From P19 onwards, we could distinguish magnocellular and parvocellular TH cells in Pac. It appears that the TH parvocellular cells are more abundant (in agreement with Swanson and Sawchenko, 1980), and some of them showed coexpression with GFP (Figures 5c–e”, 6d–e”; double-labeled parvocellular cells pointed with a filled arrowhead in panels 5e–e”, and TH single-labeled magnocellular cells pointed with an empty arrowhead in the same panels). Notably, most of the TH+ parvocellular cells of Pac coexpressed GFP at P19. Although most TH cells coexpressing GFP in Pac appear to be parvocellular, we cannot discard the existence of a few magnocellular TH cells that also coexpress GFP (as previousy suggested; Berkhout et al., 2024).

#### 3.2.3 Ventral paraventricular hypothalamic nucleus (Pav)

Regarding Pav, from preterm embryonic age E18.5 onwards, the GFP cells of this nucleus were adjacent to the classical A13 CA cell group, which contained intensely TH immunoreactive magnocellular cells. These cells partially overlapped the Pav but were mostly located laterally to it and did not coexpress GFP at any of the stages analyzed (Figures 4f, 5f, h–h”, 6h–i”, 7a–b”, 8a). Postnatally, the segregation between both became clearer, although both remained in close proximity (Figures 5f–h”, 6f–i”). At P19, extremely few and smaller TH cells were located within Pav and showed coexpression with GFP (Figures 5f–g”, 8a–b”).

**Figure 7:**
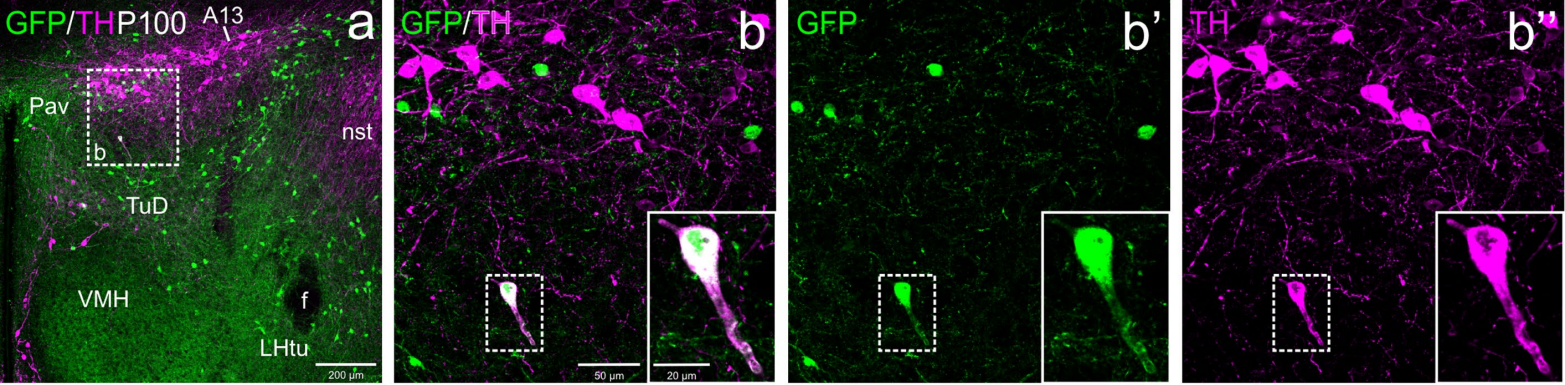
Confocal images of frontal sections of Otp-eGFP adult brains at P100, double labeled for GFP (green) and TH (magenta) (using double immunofluorescence for GFP and TH), at the level of the hypothalamic part of the zona incerta. This includes the TH+ A13 cell group, which cells do not coexpress GFP. An example of a TH/GFP double labeled cells is shown in the square in **b–b”**, which is located in the dorsal tuberal region of the basal hypothalamus. See text for more details. For abbreviations see list. Scale: a = 200 μm; b = 50 μm (applies to **b–b”**); **b–b”** insert = 20 μm.

**Figure 8:**
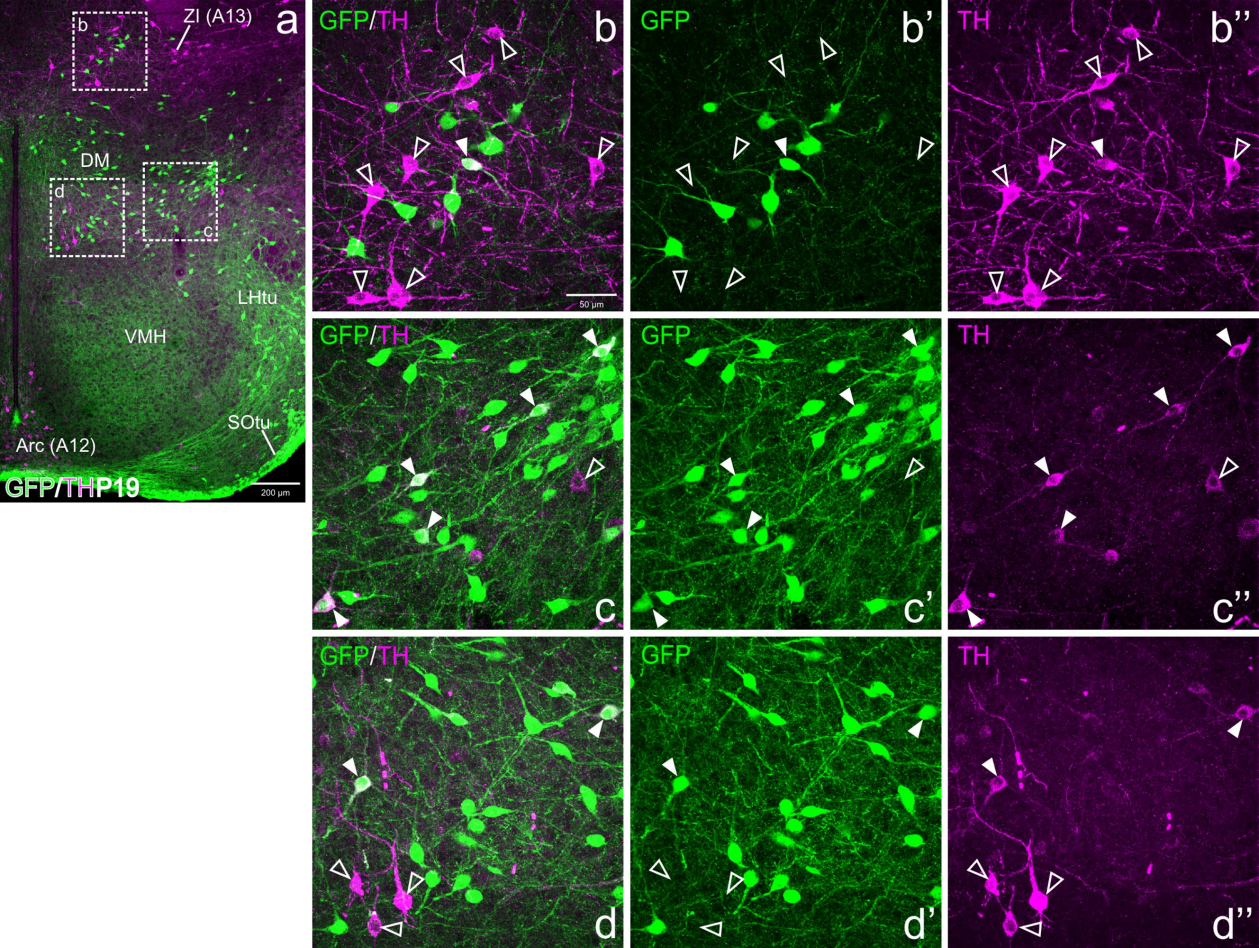
Confocal images of frontal sections of Otp-eGFP postnatal brains at P19, double labeled for GFP (green) and TH (magenta) (using double immunofluorescence for GFP and TH), at the level of the basal hypothalamus. TH/GFP double-labeled cells (filled arrowheads) and TH single-labeled cells (empty arrowheads) are seen in the dorsomedial hypothalamic nucleus (DM, details in **c–c”**, **d–d”**). See text for more details. For abbreviations see list. Scale: a = 200 μm; b = 50 μm (applies to **b–b”**, **c–c”**, **d–d”**).

#### 3.2.4 Periventricular (Pe) and subparaventricular hypothalamus (SPa)

**Figure 9:**
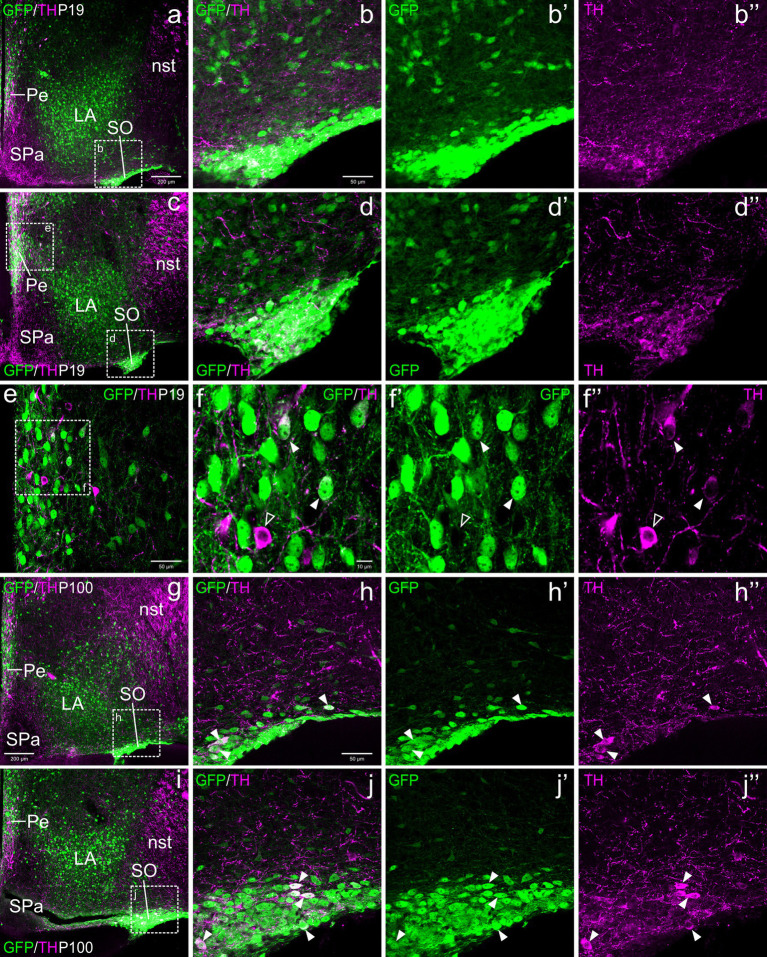
Confocal images of frontal sections of Otp-eGFP postnatal (P19) and adult (100) brains, double labeled for GFP (green) and TH (magenta) (using double immunofluorescence for GFP and TH), at different levels of the supraoptic nucleus (SO). Examples of TH/GFP cells found in this nucleus are pointed with filled arrowheads (details in **h–h”**, **j–j”**). Some double labeled cells are also found in the periventricular hypothalamic nucleus (Pe, seen in **c, e, g**), at the level of SPVco (details in **f–f”**) See text for more details. For abbreviations see list. Scale: a = 200 μm (**a, c, g, i**); b = 50 μm (applies to **b–b”**, **d–d”**, **h–h”**, **j–j”**); e = 50 μm; f = 10 μm (applies to **f–f”**).

From E18.5 onwards, we found a distinct subpopulation of TH cells in the periventricular area of the alar hypothalamus, located below the paraventricular complex, in the terminal SPVco, extending into the terminal SPa (Figures 4c, 5c,f, 6d, 9a,c,g,i). A few were also present in the suprachiasmatic nucleus (located in the terminal prosomeric part of SPa; Figure 4c). TH cells of the periventricular hypothalamic nucleus (Pe) were mostly intermingled with the GFP cells in terminal SPVco (labeled as Pe in Figures 9a,c,e,g; details in f–f”), but not in terminal SPa, where GFP cells were scarce (periventricular area of SPa indicated in Figure 9c). We found cases of TH/GFP coexpressing cells in the periventricular nucleus at terminal SPVco levels (examples of double labeled cells pointed with filled arrowheads in the details in Figures 9f–f”), but not in the periventricular hypothalamus at terminal SPa levels at any age (Figures 5c,f, 6a, 9a,c,g,i). Similarly, the TH cells of the suprachiasmatic nucleus did not co-express GFP (Figure 4c).

#### 3.2.5 Supraoptic hypothalamic nucleus (SO)

With respect to the TH cells of SO, these were observed from P19 onwards, although they represented a minor subpopulation compared to the GFP cells. All TH+ cells of SO at P19 and in adults appeared to coexpress GFP (filled arrowheads in Figures 9h–h”, j–j”).

### 3.3 Basal hypothalamus

At E18.5 and postnatally, subpopulations of TH+ cells are found in dorsal tuberal and retrotuberal areas, as well as in the dorsomedial hypothalamic nucleus (dorsal and caudal to the ventromedial hypothalamic nucleus) of the basal hypothalamus. Some cells in these areas showed co-expression of both TH and GFP (filled arrowheads in Figures 7a–b”, 8a,c–d”, 10a–b”).

**Figure 10:**
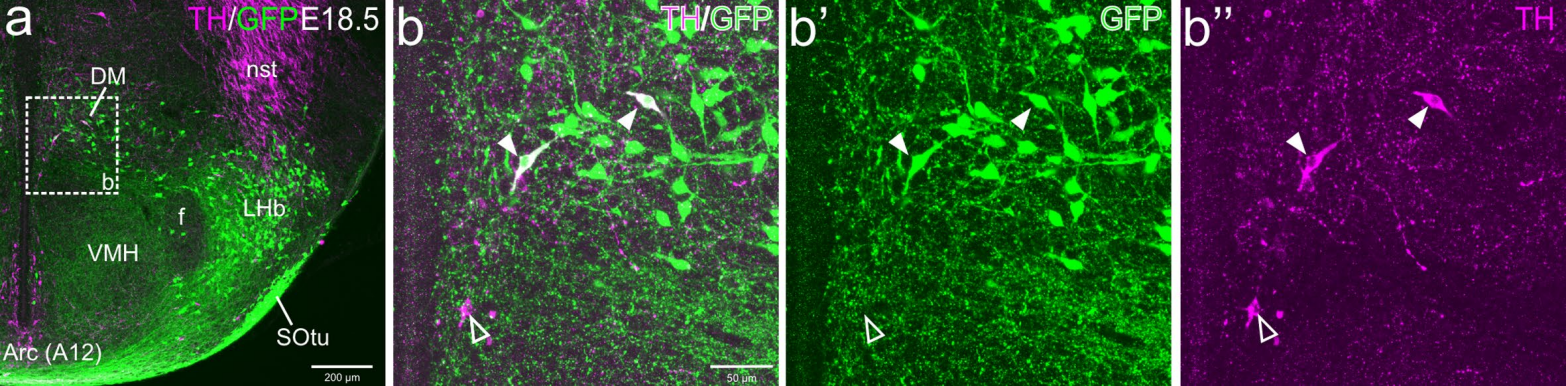
Confocal images of frontal sections of Otp-eGFP embryo brains at E18.5, double labeled for GFP (green) and TH (magenta) (using double immunofluorescence for GFP and TH), at the level of the basal hypothalamus. TH/GFP double-labeled cells (filled arrowheads) and TH single-labeled cells (empty arrowheads) are seen in the dorsomedial hypothalamic nucleus (DM, details in **b–b”**). See text for more details. For abbreviations see list. Scale: a = 200 μm; b = 50 μm (applies to **b–b”**).

## 4 Discussion

### 4.1 General findings

Our results provide evidence that some CA cells of the secondary prosencephalon belong to the Otp lineage. Using Otp-eGFP transgenic mice, we found small subpopulations of TH cells that coexpress GFP within the EAme, the periventricular preoptic area, the paraventricular hypothalamus (Pad and Pac), the periventricular hypothalamus, as well as some subdivisions of the basal hypothalamus. In some of the Otp cells, such as those of EAme, the expression of TH appears to be transitory (in agreement with previous studies; Bupesh et al., 2014), as double labeled cells are only seen at late embryonic and prepuberal stages, but not in adults. Nevertheless, this would require confirmation to discriminate between transitory expression in TH and/or GFP (due to downregulation) and cell death. In previous studies, there was a higher decrease of TH+ cells when analyzing the protein by way of immunoreactivity than when detecting the mRNA by *in situ* hybridization, which suggest that at least part of it is due to downregulation and not cell death (Bupesh et al., 2014). This transitory expression raises questions on the role of TH in the formation of specific cells and circuits of the EAme, preoptic area and hypothalamus, and in the development of specific functional systems related to homeostasis control and social behavior.

The Otp expressing cells have recently been identified as a major subtype of intermediate progenitors of the embryonic hypothalamus, that also expresses Neurog2 (Neurogenin 2), which produce Otp expressing postmitotic immature cells that differentiate into several subtypes of glutamatergic and peptidergic cells for the alar and basal hypothalamus (Zhang et al., 2021). Previous studies showed that there are different subdomains that can produce Otp-lineage cells, and the most important ones are located adjacent to the telencephalon-hypothalamic boundary, in the SPVco and the TOH (a newly identified division, previously considered a dorsal part of SPV) (for SPV: Bardet et al., 2008; García-Moreno et al., 2010; Morales-Delgado et al., 2011; for TOH: Morales et al., 2021; Metwalli et al., 2022). Considering this, it is likely that TOH/SPVco are the origin of the TH+/GFP+ cells found by us in the BSTM, the periventricular preoptic region, and the and the alar hypothalamus, as discussed in more detail below. In contrast, TH/GFP cells found in the basal hypothalamus are likely produced in the basal part of the forebrain. However, these different alar and basal areas/nuclei also contain non-Otp TH+ cells which may derive from different progenitors and co-express other transcription factors during development, as discuss below. Our results provide further evidence for the high heterogeneity of the prosencephalic catecholaminergic cell subpopulations, which is key to address better questions in order to comprehend their functions.

### 4.2 Medial extended amygdala

According to our results, double-labeled TH/GFP neurons are located in the posterior part of BSTM, but the TH immunoreactivity in the majority of these cells appeared to be transient. This agrees with previous results on the transient TH immunoreactivity in many cells of EAme, although some of the cells in BSTM continued to express the mRNA of the *Th* gene (Bupesh et al., 2014). However, in the BSTM and other parts of EAme, such as MeA and AAv, there are also some TH cells that do not co-express GFP (i.e., they do not belong to the Otp cell lineage). A previous study proposed that TH cells of EAme derive from Otp cells of SPV (Bupesh et al., 2014; see also Bardet et al., 2008; García-Moreno et al., 2010; Puelles et al., 2012), but our results based of TH/GFP coexpression showed that only some of those of BSTM may originate in this embryonic domain. Hoever, we showed that the vast majority of the Otp cells of EAme coexpress the telencephalic transcription factor Foxg1 (ranging from 91.1% in the BSTM to a 99.5% in the medial amygdala) and originates in a new telencephalic domain named the TOH, that coexpress both Otp and Foxg1 (Morales et al., 2021). Future experiments are needed to clarify if the TH cells of Otp-lineage found in BSTM do or do not coexpress Foxg1.

Regarding the TH cells of EAme that do not coexpress GFP, they may originate from other progenitors of different embryonic domain. Previous experimental evidence have identified two embryonic sources of TH+ cells in the telencephalon: the dorsal lateral ganglionic eminence (LGEd), which is a source of TH+ cells for the olfactory bulb and olfactory tubercle (Yun et al., 2003), and the commissural preoptic division (POC), which produces TH+ cells for the striatum and central extended amygdala, and possibly some of those of the preoptic area (Bupesh et al., 2014). The POC produces a subpopulation of cells for the EAme, some of which express the transcription factor (TF) Nkx2.1and the signaling protein Sonic hedgehog (García-López et al., 2008; Carney et al., 2010; Bupesh et al., 2011a). More research is needed to investigate whether this and/or other subdomains of the embryonic preoptic division produce the non-Otp TH cells seen in EAme. Single cell transcriptome data in mouse has identified a subpopulation of TH postmitotic cells of the preoptic area that coexpress the TFs Lhx6 and Gbx1, which derives from intermediate progenitors expressing Ascl1 in combination with Gsx1 and Islet1 (Zhang et al., 2021). Cells expressing some of these TFs (Lhx6, Gbx1, Islet1) have been found in the subventricular zone and mantle of the preoptic embryonic division, and some (Lhx6, Islet1) were also found in the medial extended amygdala in mouse and chicken (García-López et al., 2008; Abellán and Medina, 2009; Bupesh et al., 2011b; Vicario et al., 2015). Since the Ascl1 progenitors are a source of GABAergic cells (Zhang et al., 2021), it is likely that the TH+ cells of Ascl1-cell lineage are also GABAergic, which is in line with previous data showing that the subpallium mainly produces GABAergic neurons (Abellán and Medina, 2009; Osório et al., 2010) and showing expression of GABAergic markers in TH+ cells (Romanov et al., 2017; Negishi et al., 2020). In addition, we cannot discard that the non-Otp TH cells of the medial amygdala and AAv originate in TOH and/or SPV but from non-Otp progenitors. In this sense, it is important to remember that, although the majority of Otp cells in SPV (including the TOH) coexpress Sim1, a small group of cells only express Sim1 without Otp, giving rise to subpopulations of cells which differentiation is independent of Otp (Acampora et al., 2000). Further investigation is needed to clarify this question.

### 4.3 Preoptic area

Our results showed that some TH cells of the periventricular preoptic area coexpress GFP, although non-GFP TH+ cells are also found in this area. These two different types of TH cells might derive, respectively, from intermediate progenitors expressing Otp/Neurog2 (mainly those of TOH) and from progenitors expressing Ascl1 and other TFs (references above). However, in the periventricular preoptic area, most TH cells might derive from Otp progenitors, as only 13% of them coexpress GABAergic markers (Negishi et al., 2020). Interestingly, previous studies in rats showed that the anteroventral periventricular preoptic area is sexually dimorphic, with a larger volume in females, and contains TH cells that are more abundant in females than in males, are sensitive to sex steroid hormones during development and in adulthood, and play a role in regulation of estrous cycle in sexually mature females (Simerly et al., 1985a, 1985b; Simerly, 1989; Orikasa et al., 2002). Additional studies are needed to investigate which of the two TH+ cell subpopulations of the periventricular preoptic area, regarding their embryonic origin, are involved in the regulation of the estrous cycle in females, and what is the functional relationship between both subtypes and with other neurons of the SBN.

While most sexually dimorphic TH cells of the periventricular preoptic area are located ventrally (Simerly, 1989; Orikasa et al., 2002), we also observed TH cells in the dorsal periventricular preoptic area, in relation to the POC division (as also described by Bupesh et al., 2014). The latter TH cells extended laterally to the vicinity of BSTMv, where it formed a distinct subgroup of TH cells within the parastrial nucleus, which could be better observed at P19 and in adults. This nucleus contains cells coexpressing TH and GFP at P19, although not later. The TH cells of this nucleus appear to overlap a distinct subpopulation of cells expressing oxytocin and/or vasopressin (identified as AC or AC/ADP by Otero-Garcia et al., 2014, Otero-García et al., 2016). The latter cells appear to derive from Otp-lineage cells of the SPV domain (Wang and Lufkin, 2000; discussed by Morales et al., 2021) and this could also be true of the TH+ Otp cells found in this nucleus. It remains to be known if there is at least partial coexpression between TH, oxytocin and vasopresin in the same cells, as shown in the alar hypothalamus by single-cell transcriptome (Romanov et al., 2017).

### 4.4 Paraventricular, periventricular and supraoptic nuclei of the TOH and alar hypothalamus

Regarding the paraventricular nuclear complex, we found that a part of the TH neurons of the Pad (anterior) and Pac (central Pa) coexpressed GFP, whereas most of those of the Pav (posterior) and adjacent A13 group did not. Our results on the presence of Otp TH neurons in Pac agree with those of Berkhout et al. (2024) based on single-cell transcriptomics. Moreover, some TH/GFP double labeled cells were located in the SO, in both its terminal and peduncular (main) subdivisions. The Otp cells of Pad primarily originate within the peduncular part of TOH and express the transcription factor Foxg1, while those of Pac, Pav and SO primarily originate in SPVco and do not coexpress Foxg1 (Morales et al., 2021). Based on this, it is likely that Otp TH cells of Pad originate in TOH, while those of Pac and SO originate in SPVco. Nevertheless, we cannot discard the existence of cellular interchange between TOH and SPVco due to tangential migrations along the dorsoventral axis (described previously based on coexpression with Foxg1 in Otp cells, although they appear to be scarce; Morales et al., 2021; Metwalli et al., 2022). Therefore, analysis of Foxg1 is necessary to clarify this issue. Although many of the TH cells of Pad, Pac and SO coexpressed GFP and belonged to the Otp-lineage, some TH cells in these nuclei did not, raising questions about the embryonic origin of these neurons.

A surprising finding of our study is that most of the TH cells located in the Pav did not co-express GFP and, apparently, do not derive from Otp cell progenitors. The embryonic origin of these cells is intriguing. One possibility is that they originate from cells of the ventral SPVco that express Sim1 but not Otp. Alternatively, they may derive from non-Otp progenitors of the basal hypothalamus, as it is likely the case for the TH+ dopaminergic neurons of A13 (Bilbao et al., 2022). In particular, the A13 CA cell group might originate in the retrotuberal dorsal hypothalamic area (RtuD) of the basal hypothalamus (Bilbao et al., 2022), a region characterized by the expression during development of different transcription factors such as Nkx2.1, Ascl1, Islet1, Otp and Sim1 (Diaz and Puelles, 2020; for Ascl1 see Allen Developing Mouse Brain Atlas).

### 4.5 Basal hypothalamus

As noted above, the dorsal RTu region of the basal hypothalamus contains the A13 CA cell group, that do not belong to the Otp-lineage. However, we found that some of the TH+ cells coexpressed GFP in dorsal and more ventral parts of the RTu, including the dorsomedial hypothalamic nucleus (DMH), which is involved in control of metabolism and thermoregulation, but is also related to the social behavior network (SBN), becoming active during social defeat stress (Kataoka et al., 2014; Nakamura et al., 2022). It is unclear whether the TH+ Otp cells migrate from SPV or from the periretromamillary area, which also expresses Otp (Morales-Delgado et al., 2011). However, the latter area is rather poor in TH+ cells (Bilbao et al., 2022), making this a less likely source of the TH Otp cells found in RTu and DMH. Clarifying the origin of these cells is important, as it can help to better understand other features of these cells, including their specific connections and function. Regarding the non-Otp TH+ cells, they likely originate from progenitors expressing Nkx2.1, Ascl1 and/or Islet1, which have been involved in producing GABAergic cells (as explained above).

### 4.6 Function of the CA cells of the social brain network (SBN)

A major finding of our study is that there are two major subtypes of TH+ cells, Otp-related and non-Otp TH cells, distributed throughout many of the centers of the SBN, including the medial extended amygdala, preoptic area and hypothalamus. Many of these forebrain centers described here that contain different subtypes of TH cells are interconnected and are involved in regulation of different aspects of social behavior (Canteras et al., 1995; Newman, 1999; Dong and Swanson, 2004, 2006; Abellán-Álvaro et al., 2022; reviewed by Chen and Hong, 2018).

The role of catecholamines, in particular dopamine, has been mainly studied in the mesocorticolimbic system, in relation to social reward, but dopamine also plays a role in modulation of the social brain network (Young and Wang, 2004; Young et al., 2011; Nowicki et al., 2000). The role of the latter in pair bond formation and mating has been studied in monogamous species of different vertebrates, from fishes to mammals (Nowicki et al., 2000). The function of the SBN is modulated by dopamine receptors 1 and 2 (Nowicki et al., 2000). The areas/nuclei of the SBN are innervated by dopaminergic fibers of extrinsic origin, possibly including inputs from the ventral tegmental area (Hasue and Shammah-Lagnado, 2002), but many of the centers of this network also contain subpopulations of CA neurons, as explained above.

In murines, the involvement of the CA neurons in aspects of sexually-dimorphic social behavior has been demonstrated for those of AVPV in the preoptic area (Simerly et al., 1985a; Scott et al., 2015). However, CA cells of EAme are very scarce in murines, and expression of TH appears to be transitory (Northcutt et al., 2007; Bupesh et al., 2014), which have hinder studies on their function in these species.

Knowledge on the role of the TH cells of the medial extended amygdala mainly comes from studies in the monogamous prairie vole, as hundreds of TH+ cells are present in the medial amygdala and the BSTM in adult animals (Northcutt et al., 2007; Northcutt and Lonstein, 2009, 2011; Ahmed et al., 2012). In both the BSTM and medial amygdala of prairie voles, the number of TH+ cells is much larger in males than in females (Northcutt et al., 2007). It appears that many TH+ cells of the medial amygdala and BSTM of prairie voles project to the medial preoptic area (Northcutt and Lonstein, 2011), a center very important for socioreproductive behavior (Newman, 1999). The projections include the anteroventral periventricular preoptic area (AVPV), which also includes TH+ cells that show sexual dimorphism in murids (Simerly, 1989) but not in the monogamous prairie voles (Lansing and Lonstein, 2006; Ahmed et al., 2012). Since these cells are more numerous in rat females and have been involved in regulation of estrous cycle (Simerly et al., 1985a, 1985b; Simerly, 1989), it has been suggested that the lack of dimorphism in prairie voles is related to the fact that prairie vole females are induced ovulators, instead of spontaneous ovulators like the rat (discussed by Ahmed et al., 2012).

One intriguing aspect of the TH neurons of the medial amygdala and BSTM of prairie voles is that they only appear to produce L-DOPA, as they lack the enzyme AADC, necessary for the synthesis of dopamine (Ahmed et al., 2012). The TH+ cells of these nuclei project to the medial preoptic nucleus (POM), where most of the CA cells express AADC but not TH (i.e., they are TH-/AADC+), although the POM also contains TH+/AADC+ cells (Ahmed et al., 2012). It has been suggested that the TH+/AADC− cells of the EAme and POM might work in cooperation with the TH-/AADC+ cells to produce dopamine (Ahmed et al., 2012), as suggested elsewhere (Nowicki et al., 2000). Nevertheless, L-DOPA released from terminals can directly interact with dopamine receptors (Viaro et al., 2021), and might also modulate social behavior directly. Most TH+ cells of AVPV also lack AADC in prairie vole, and extremely few or no cells are immunoreactive for both TH and AADC. It would be necessary to analyze TH and AADC coexpression in cells of EAme and preoptic area in mouse, and study how different cells regarding TH/AADC coexpression relate to the Otp versus non-Otp lineage. Moreover, this also raises questions about the role of TH in some of the cells of EAme and preoptic area during development, and why expression decays around puberty.

The EAme (including medial amygdala and BSTM) also projects to the paraventricular, periventricular and supraoptic nuclei of TOH and alar hypothalamus, where Otp and non-Otp cells are present. In contrast to those of EAme, the TH+ cells of these nuclei increase the expression of this enzyme with age. Most TH cells of the paraventricular nucleus are parvocellular (in agreement with previous studies; Swanson and Sawchenko, 1980) and the majority of them belong to the Otp-cell lineage. This appears to be similar in the SPV-part of the periventricular hypothalamic nucleus. In contrast, the TH+ cells of the SPa-part of the periventricular hypothalamus are mostly magnocellular and do not belong to the Otp-lineage. The paraventricular, periventricular and supraoptic nuclei of the hypothalamus contain subpopulations of neurons expressing vasopressin and/or oxytocin, long known for their role in neuroendocrine control (through projections to the neurohypophysis) but also social affiliation, reward and pair-bonding (through projections to mesolimbic and EAme nuclei) (De Vries and Miller, 1998; Walum and Young, 2018). Mouse single cell transcriptome data indicate that vasopressin and/or oxytocin cells may express TH at very low levels (Romanov et al., 2017; see also Berkhout et al., 2024), and coexpression of TH and oxytocin has been shown to occur in a subset of neurons of the supraoptic nucleus in humans (Panayotacopoulou et al., 1994, 2000; Kontostavlaki et al., 2007). It appears that a subpopulation of dopaminergic parvocellular neurons of the paraventricular alar hypothalamus projects to the median eminence (Ferguson et al., 2008) and many dopaminergic cells of this nucleus express kisspeptin receptors, suggesting that they may be involved modulation of reproduction (Higo et al., 2021; Grassi et al., 2022). Regarding the dopaminergic cells of the periventricular alar (anterior) hypothalamus (Pe), they receive inputs from the suprachiasmatic nucleus that regulate their circadian cycle of activation, and these CA neurons project to the lateral septum to regulate locomotion (Korchynska et al., 2022). The activity of the suprachiasmatic neurons as well as their target CA neurons of Pe increases during the dark hours in mice (Korchynska et al., 2022). Considering that Pe of the alar hypothalamus contains Otp and non-Otp CA cells, it would be important to study which subtype is involved in this pathway. Moreover, based on single cell transcriptomics (Romanov et al., 2017; Zhang et al., 2021), possibly the first subtype is glutamatergic and the second subtype GABAergic, making a big difference regarding their function.

## Data Availability

The original contributions presented in the study are included in the article/supplementary material, further inquiries can be directed to the corresponding author.

## Glossary

A13: A13 catecholaminergic cell group
AA: Anterior amygdala
AAv: Ventral AA
ac: Anterior commissure
ACo: anterior cortical amygdalar area
acp: Posterior limb of the anterior commissure
AH: Anterior hypothalamic area
Arc: Arcuate nucleus
AVPV: Anteroventral periventricular preoptic nucleus
BST: Bed nucleus of the stria terminalis
BSTM: Medial BST
BSTMp: BSTM, posterior division
BSTMv: BSTM, ventral division
BSTL: Lateral BST
BSTLv: BSTL, ventral division
BSTLp: BSTL, posterior division
DM: Dorsomedial hypothalamic nucleus
EAme: Medial extended amygdala
f: Fornix
GFP: Green fluorescence protein
IPAC: Interstitial nucleus of the Pac
LA: Lateral anterior hypothalamic area
LH: Lateral hypothalamus
LHb: Basal part of LH
LHtu: Tuberal LH
LPo: Lateral posterior hypothalamic area
LPO: Lateral preoptic area
Me: Medial nucleus of the amygdala (or simply, medial amygdala)
MeA: Anterior subnucleus of Me
MeP: Posterior subnucleus of Me
MePD: Dorsal subnucleus of MeP
MePV: Ventral subnucleus of MeP
nst: Nigrostriatal tract
Pa: Paraventricular hypothalamic nucleus
pac: Posterior limb of the anterior commissure
Pac: Central Pa
Pad: Dorsal Pa
Pav: Ventral Pa
Pavl: Ventrolateral Pa
Pe: Periventricular hypothalamic area
PO: Preoptic area
SCN: Suprachiasmatic nucleus
SEA: Sublenticular extended amygdala
SPa: Subparaventricular region
SPVco: Supraopto-paraventricular hypothalamic domain, core part
SO: Supraoptic nucleus, principal subdivision
SOtu: Supraoptic nucleus, tuberal subdivision
TH: Tyrosine hydroxylase
TOHp: Telencephalo-opto-hypothalamic embryonic domain, peduncular subdivision
TOHt: Telencephalo-opto-hypothalamic embryonic domain, terminal subdivision
TuD: Dorsal tuberal area
VMH: Ventromedial hypothalamic nucleus
ZI: Zona incerta
